# Early COVID-19 response in two small island developing states: Maldives and Trinidad and Tobago

**DOI:** 10.5365/wpsar.2022.13.1.885

**Published:** 2022-03-31

**Authors:** Shalini Pooransingh, Abdul Azeez Yoosuf, Sheena Moosa, Nishan Ahmed, Satish Jankie, Lexley Pinto Pereira

**Affiliations:** a Faculty of Medical Sciences, The University of the West Indies, St Augustine, Trinidad and Tobago.; b Private physician, Malé, Maldives.; c Research Development Office, The Maldives National University, Malé, Maldives.; d Health Protection Agency, Malé, Maldives.

## Abstract

**Problem:**

Coronavirus disease 2019 (COVID-19) was declared a pandemic on 11 March 2020. Severe illness requires intensive care facilities, which are limited in smaller, resource-constrained settings.

**Context:**

Maldives and Trinidad and Tobago are small island developing states with comparable climates. Similar to island nations in the Western Pacific Region, they are prone to natural disasters and so engage in planning and preparedness activities on an ongoing basis. This paper describes the initial measures taken by both countries during the first wave of COVID-19, from March to May 2020.

**Action:**

In both countries, multisectoral high-level leadership allowed for timely and decisive actions. Early school closures, early border closures and early lockdowns were enforced. Mandatory mask wearing and physical distancing were instituted. Cases and contacts were isolated in facilities away from public sector hospitals, and isolation was implemented at the government’s expense. Volunteers were trained to manage dedicated hotlines. Additionally, the governments held daily press briefings.

**Outcome:**

During the first wave, Maldives contained its epidemic to one geographical cluster; Trinidad and Tobago successfully avoided community spread, thus averting an overwhelmed health system.

**Discussion:**

Diligent contact tracing with quarantine implemented at the government’s expense successfully minimized spread in both countries. Small countries need volunteers to help with activities such as contact tracing, and recruiting and training volunteers before a health emergency occurs is key. Lessons learned from the experience of Maldives and Trinidad and Tobago could serve as a model for other small island developing states, including those in the Western Pacific Region.

## Problem

The World Health Organization (WHO) declared coronavirus disease 2019 (COVID-19) a pandemic on 11 March 2020. (*1*) Severe COVID-19 may require intensive care, which is limited in smaller, resource-poor nations. This paper describes how two small island nations managed the first wave of the pandemic. Their handling of the pandemic during the first 3 months contained the spread. The lessons learned from their experience may assist small island developing states (SIDS) in WHO’s Western Pacific Region.

### Context

Maldives and Trinidad and Tobago are SIDS. (*2*) Maldives has a population of 437 535 persons living on 187 islands in the Indian Ocean (*3*) and has a human development index score of 0.719. (*4*) Total health expenditures are 9.0% of gross domestic product. (*5*) Trinidad and Tobago, in the Atlantic Ocean, has a population of 1.4 million persons and is also high on the human development index, with a score of 0.799. (*4*) Total health expenditures are 7.0% of gross domestic product. (*5*) These countries were chosen for this study because they are small island states with relatively high scores on the human development index. They have similar climates, and both countries have experienced natural disasters and are engaged in ongoing planning and preparedness activities. They are similar to SIDS in the Western Pacific Region, such as Fiji, (*6*) in that tourism is a major income generator for Maldives and to a lesser extent for Trinidad and Tobago, and both depend on imports for many items.

This paper describes the initial measures taken by Maldives and Trinidad and Tobago during their first waves of COVID-19 (from 7 March in Maldives and 12 March in Trinidad and Tobago to 31 May 2020) that helped keep their health systems from being overwhelmed. The descriptive reports on which this paper is based were made at each country’s government briefings.

The first case of COVID-19 in Maldives was detected at a tourist resort on 7 March 2020; by 14 April, there were 20 confirmed imported cases, with 56 suspected cases in isolation and 152 contacts under quarantine. The first local case detected on 15 April in the capital, Malé, prompted a lockdown of the greater Malé area. Cases increased during the following weeks, and by  31 May 2020, there were 1730 cases, 127 recoveries, 12 hospitalizations and 5 deaths. (*7*)

Trinidad and Tobago reported its first confirmed case of COVID-19 on 12 March 2020 in a traveller returning from Europe. On 13 March, a second case was confirmed, and by 19 March, nine cases were confirmed. On 21 March, 40 cases were confirmed in a group of 68 returning nationals who were aboard a cruise liner. The first death from COVID-19 in Trinidad and Tobago occurred on 25 March, and by 31 May, there were 117 cases and 8 deaths. (*8*)

In the early phase, the majority of cases in Maldives occurred among males (> 80%; 1412/1730). In Maldives, most cases were aged 21–40 years, while in Trinidad and Tobago, the majority of cases were aged 60–69 years. In Maldives, the majority of cases occurred among foreign migrant workers (65%; 1124/1730), with one third observed among Maldivians. In Trinidad and Tobago, most cases were imported, with 34% (40/117) occurring in one cluster on a cruise liner.

### Action

In Maldives, the Health Protection Agency is mandated to safeguard the nation’s public health. The Director of Public Health heads the Agency and reports to and advises the Health Minister.

In Trinidad and Tobago, County Medical Officers of Health are the public health officers in charge of each county’s surveillance and response activities. They report to the Chief Medical Officer at the Ministry of Health.

Both countries adopted a multisectoral approach. The early actions taken by both countries during their first wave of COVID-19 resulted in Maldives containing the epidemic to one geographical cluster, while Trinidad and Tobago managed to avoid community spread, thus preventing its health system from becoming overwhelmed.

### Lessons learned

#### Multisectoral high-level leadership led to a timely response

In Maldives, the Health Emergency Coordination Committee, activated on 21 January 2020, coordinated the multiagency response. As the situation escalated, response coordination was handed to the National Disaster Management Authority to facilitate multiagency coordination; health emergency operations were relocated to the National Emergency Operations Centre on 1 March 2020. With this change, a national task force was established at the executive level, and the strategic decision-making body was chaired by the President. A national public health emergency was declared on 12 March 2020.

In Trinidad and Tobago, a core team was formed to manage the response, comprising the Minister of Health, the Minister of National Security and the Chief Medical Officer and his team of public health professionals at the ministry and county levels. Communication was at the forefront of the response.

In both countries, communication about the pandemic came from trusted sources. In Maldives, communication was led by the President’s office, which held daily press briefings; multiskilled staff from across the government and private sectors facilitated content production and managed communication through mass and social media. At the National Emergency Operations Centre, the call centre was operated by dedicated personnel and a large number of volunteers.

In Trinidad and Tobago, the Minister of Health and the Chief Medical Officer, along with subject matter experts, held daily press briefings that included clinical and epidemiological updates and information about the availability of testing facilities. Potential mental health challenges from job loss, domestic violence and stay-at-home restrictions were addressed by clinical psychologists. A hotline staffed by doctors was established as a resource for the public.

#### Preparedness activities were initiated early and focused on points of entry

In both countries, preparedness activities were initiated approximately 6 weeks before WHO declared the pandemic. Early interventions focused on points of entry. Trinidad and Tobago began its prevention and response efforts on 23 January 2020, implementing entry and exit screening at airports. In Maldives, the early response focused on the tourism sector and travellers to facilitate early detection, contact tracing and isolation. Although evidence supporting entry and exit screening is sparse, these activities can earn public confidence and raise awareness among the travelling public. (*9*) In retrospect, the asymptomatic phase of the disease means that such individuals would have been missed during airport screening. Border closures were implemented early in both countries, and as in several countries in the Western Pacific Region, this measure was successful in limiting the entry of the virus and keeping case numbers low.

#### Public health and social measures were initiated early and adjusted as needed

**Fig. 1:**
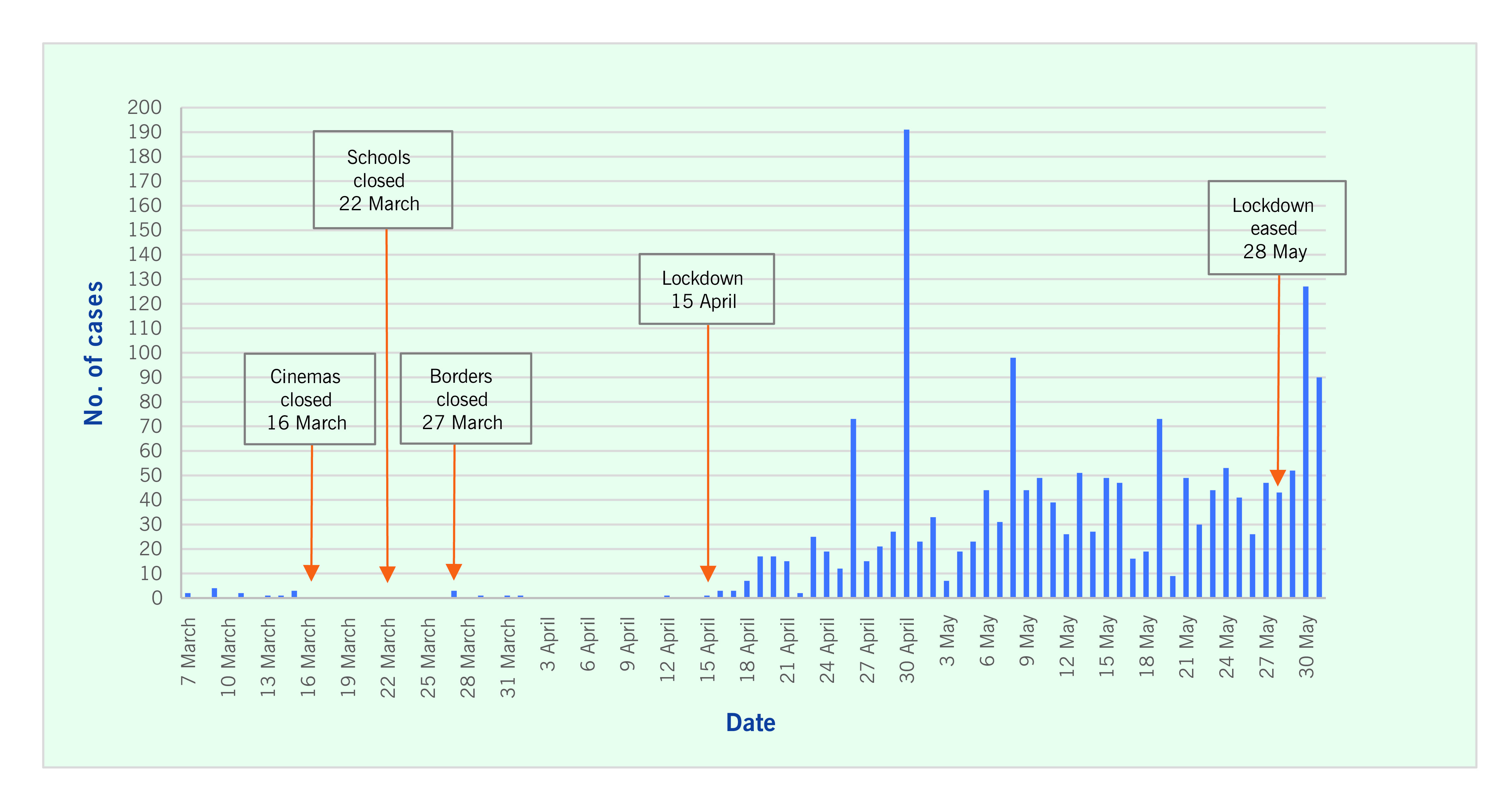
Epidemic curve of COVID-19, Maldives, 7 March to 31 May 2020

**Fig. 2:**
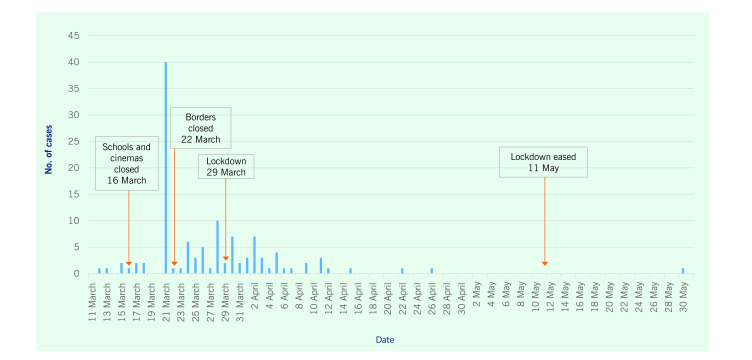
Epidemic curve of COVID-19, Trinidad and Tobago, 11 March to 31 May 2020

Cinemas and educational institutions were closed when there were 13 cases in Maldives and 5 in Trinidad and Tobago. Borders were closed when Maldives reached 14 cases and Trinidad and Tobago reached 10. Lockdown of non-essential services occurred on 15 April in Maldives and on 29 March in Trinidad and Tobago. Lockdown in the latter included the closure of restaurants and a rapid scaling down of public gatherings from 25 to 5 persons; residents were permitted to visit only supermarkets, pharmacies and hardware stores (**Table 1**; **Fig. 1** and **2**). In Maldives, lockdown measures were eased on 28 May, but physical distancing and mask wearing were made mandatory for essential services and their providers (**Table 1**; **Fig. 1**). In Trinidad and Tobago, face masks became mandatory in public places on 5 April, based on international guidance. (*10*) On 11 May, lockdown measures were eased with a “no mask, no service” rule in effect (**Table 1**; **Fig. 2**).

[**insert** Figure 1]

[**insert** Figure 2]

**Table 1:** Timeline of public health and social measures and number of cases of COVID-19, Maldives and Trinidad and Tobago, March–May 2020

Measures	Maldives		Trinidad and Tobago	
	Date	No. of cases	Date	No. of cases
First case detected	7 March (imported)	1	12 March (imported)	1
Closure of cinemas	16 March	13	16 March	5
Closure of all educational institutions	22 March	13	16 March	5
Borders closed	27 March (suspension of on-arrival visas)	14	22 March (closure includes returning nationals)	10 (plus 40 on a cruise ship)
Lockdown of non-essential businesses and services, with enforcement	15 April	21	29 March	38 (plus 40 on a cruise ship)
Easing of phase 1 lockdown	28 May	1528	11 May	116

In Maldives, in the early stages of the pandemic, community-based interventions and health risk communication did not have the expected impact on physical distancing despite the closure of educational institutions and some workplaces on 22 March because the contact tracing of the first community case identified more than 100 close contacts. (*11*) This led to enforced movement restrictions and scaling up of risk communications. As a result, within 3 weeks of lockdown and enforcement, contact bubbles were reduced to eight persons per case. This contrasts with Trinidad and Tobago where enforcement of public health measures accompanied lockdown early in the epidemic (18 days after the first case), with citizens allowed outside only to visit supermarkets, pharmacies and hardware stores. Community spread was not reported in Trinidad and Tobago at that time.

#### Multiagency action supported contact tracing in the containment phase

Surveillance with contact tracing and case investigation was supported by rapid response teams in Maldives and by county office staff in Trinidad and Tobago. In both countries, additional health-care workers, volunteers and students were recruited and trained for contact tracing. Contact tracing was prioritized in Maldives, with near-universal contact tracing within 48 hours of case detection and cases isolated within 2 days. Multiagency action supported by information technology allowed for speedy implementation of containment activities. The use of volunteers in both countries suggests that volunteers should be recruited and trained before a health emergency occurs. (*12*)

#### Isolation at state expense likely delayed community transmission

In Trinidad and Tobago, patients were isolated in facilities away from public sector hospitals and private nursing homes, and the government established two facilities with intensive care units (ICUs) and resources for high-dependency care. Health workers caring for patients were housed separately. Suspected cases were isolated in facilities at the government’s expense. Discharge from quarantine required two negative test results 24 hours apart.

In Maldives, an ICU ward at the national referral hospital was designated to care for severe cases. One private hospital was commissioned to manage severe cases while work progressed to establish a separate hospital facility for these cases. Quarantine and isolation facilities were established at resort islands managed by the National Emergency Operations Centre. Suspected cases in the community were isolated for monitoring in specially designated facilities. Suspected cases were provided with food and essential commodities and supported by the Maldives Red Crescent.

In both countries, isolation of suspected cases in specially designated facilities at the government’s expense, rather than at home, likely contributed to containment of the outbreaks. (*13*)

#### Laboratory testing supported the response

In Maldives, strengthening laboratory capacity required activating the emergency contingency budget and mobilizing aid resources to obtain materials for testing. The capacity for polymerase chain reaction testing was increased from 200 tests per day to 1000 tests per day in May 2020. Laboratories in the private sector and the forensic laboratory at Maldives Police Services were mobilized. Genomic sequencing is available through regional reference laboratories.

Trinidad and Tobago followed WHO protocols (*14*) for laboratory testing in April 2020: testing was widened to include frontline personnel in public sector facilities and elderly care facilities, and sentinel surveillance for influenza-like symptoms was implemented. During the initial phases, genomic sequencing was locally available only through the University of the West Indies St Augustine Medical School.

Box 1 **highlights key lessons learned during the early response in both countries.**

**Box 1. Lessons learned**

- Early lockdown requires enforcement if it is to be effective at preventing and containing the spread of disease.
- Strong health communication is essential: communities need to be made aware of the rationale for a lockdown and should be engaged in the response to the pandemic.
- Allowing people to isolate in a facility at the government’s expense appeared to contain the spread during the first wave of COVID-19.
- Small countries may lack the human resources necessary to carry out sustained public health activities, such as contact tracing, implementing a rapid response and staffing hotlines. Volunteers are a useful resource for these public health activities; potential volunteer pools should be identified.
- Taking a regional approach to guarantee laboratory access and vaccine procurement would ensure equitable access for smaller nations.

## Discussion

Prompt action was critical to contain the COVID-19 pandemic early in both countries. Keeping the numbers low is important for nations with limited resources and less sophisticated health-care systems to buy time until vaccines and medications become available.

Since the first wave of COVID-19 (March to May 2020), both countries have experienced further waves. As of the time of writing, Maldives experienced its latest surge in September 2021, and Trinidad and Tobago is in its fourth wave, with a surge in cases. Trinidad and Tobago was under a prolonged state of emergency from 16 May to 17 November 2021. From 4 October, fully vaccinated persons were once again allowed to visit cinemas and gyms, and schools reopened to fully vaccinated students who were in their past 4 years of secondary school. In Maldives, school reopening has been staggered, with older students returning first, such as those sitting examinations.

In Trinidad and Tobago, vaccination began in February 2021 with donated vaccines and the prioritization of health-care workers. In April 2021, those aged (*3*)60 years and those with comorbidities were prioritized, and by August 2021, vaccination was open to all persons aged (*3*)12 years, including persons from migrant communities. Trinidad and Tobago has achieved 46.3% full vaccination coverage of its eligible population.

In Maldives, vaccination commenced in February 2021 for those aged (*3*)65 years, persons with comorbidities and frontline workers (including foreign migrant workers). By April 2021, vaccination was available to everyone aged (*3*)18 years, including foreign migrants. Maldives has achieved 67.5% full vaccination coverage of its eligible population.

The responses of both countries during their first waves served to limit spread and prevent their health systems from being overwhelmed. Lessons may also be derived from the management of the pandemic in the Pacific Island SIDS that closed their borders early. Leaders in the Pacific Islands Forum invoked the Biketawa Declaration to mount a collective response to the pandemic. Additionally, a Pacific action COVID-19 preparedness and response plan was developed to reduce transmission and to manage cases. The plan included activities such as screening passengers at major checkpoints, implementing 14-day quarantine for contacts and closing entry to non-residents. It also included the sharing of resources among islands. The Pacific Humanitarian Pathway on COVID-19 is the Region’s mechanism for enabling the political commitment to expedite assistance and foster cooperation among member countries. It also facilitates the provision of medical and humanitarian assistance from regional, international and development partners in a timely and equitable manner. (*15*, *16*)

In conclusion, ongoing planning and preparedness, multisectoral collaboration, and community engagement and participation are critical to ensuring a successful response to an outbreak such as COVID-19. (*17*, *18*)
